# A Rare Case of Traumatic Colonic Intramural Hematoma in Saudi Arabia

**DOI:** 10.7759/cureus.51461

**Published:** 2024-01-01

**Authors:** Hussain S Ibraheem, Masooma S Hashem, Sara H Ebrahim, Manal M Alshehab, Zahra H Alali, Ali A Alhayki

**Affiliations:** 1 Surgery, Qatif Central Hospital, Dammam, SAU; 2 Medicine, King Fahad University Hospital, Imam Abdulrahman Bin Faisal University, Al-Khobar, SAU

**Keywords:** conservative, trauma, ct with contrast, intramural hematoma, colon

## Abstract

Colonic intramural hematomas are rarely encountered clinically. They are most commonly caused by blunt trauma to the abdomen. Diagnosis is usually reached by a combination of a detailed history, physical examination, and radiological investigations.

A 14-year-old female patient presented to the emergency department complaining of abdominal pain with a history of a go-karting accident. Upon physical examination, the patient was tachycardic and hypertensive, with right-side abdominal tenderness and fullness. After going through routine radiological investigations, a computed tomography scan showed a large intramural hematoma of the ascending colon measuring around 7.7 x 8.4 x 2 cm. The patient was admitted for conservative management. Throughout her admission, serial examinations were performed, which showed improvement in the patient’s condition and the size of the hematoma. The patient was discharged in a stable condition after showing good recovery. Following up with the patient a month later, she was in good condition with no active complaints, and an ultrasound was done that revealed complete resolution.

To our understanding, this report of colonic intramural hematoma caused by the unusual etiology of the go-karting accident, which was successfully managed conservatively, adds significantly to the literature.

## Introduction

Colonic intramural hematoma is a rare clinical condition, accounting for about 4.4% of all gastrointestinal intramural hematomas [1], with only a few cases being reported in the world medical literature. It is most commonly caused by trauma, anticoagulation therapy, and hemorrhagic diseases [1,2]. Colonic intramural hematoma is diagnosed based on clinical history, physical examination, and imaging modalities such as conventional radiography, ultrasonography (US), computed tomography (CT), and colonoscopy (CS) [3]. The optimum treatment should be tailored according to the patient’s clinical condition [4]. We herein report a case of traumatic intramural hematoma that was treated conservatively with a good recovery.

## Case presentation

On the 19th of June 2022, a 14-year-old female patient, medically free, presented to the emergency department complaining of severe intermittent right hypochondrial abdominal pain with a score of 9 out of 10 that was worse with eating and deep breathing and improved with paracetamol. The patient was tolerating oral intake with normal bowel motions. The patient's symptoms commenced after she hit a still object on the right side of the abdomen while she was go-karting one week earlier. At that time, the patient was admitted to the intensive care unit (ICU) in a private hospital on the 12th of June 2022 due to a rapid drop in hemoglobin. A CT was performed at that time, which showed an intramural ascending colon hematoma measuring 12.5 x 9.5 x 9.5 cm (AP x TRV x CC, respectively).

**Figure 1:**
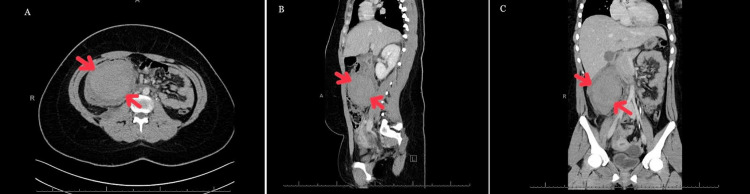
Abdominal contrast-enhanced CT images (A) Axial view revealed a hyperdense mass in the ascending colon (white arrow). (B) Sagittal view showing a hematoma at the ascending colon (white arrow). (C) Frontal view

On physical examination in the current presentation, the patient was tachycardic with a heart rate of 160 beats per minute and hypertensive with a blood pressure of 197/102. The chest examination was unremarkable. Abdominal examination revealed marked tenderness and guarding at the right hypochondrial area, swelling, and no rigidity with normal bowel sounds. Laboratory investigations revealed moderate anemia (10.9 g/dl) (reference range: 11.4-14.7), and the coagulation profile and chemistry were within the normal range. Radiological investigations include extended focused assessment with sonography in trauma, and chest and abdominal X-rays were unremarkable. An abdominal CT with contrast was completed and confirmed the presence of a large intramural hematoma of the upper part of the ascending colon and hepatic flexure of 7.7 x 8.4 x 2 (Figure 1).

**Figure 2:**
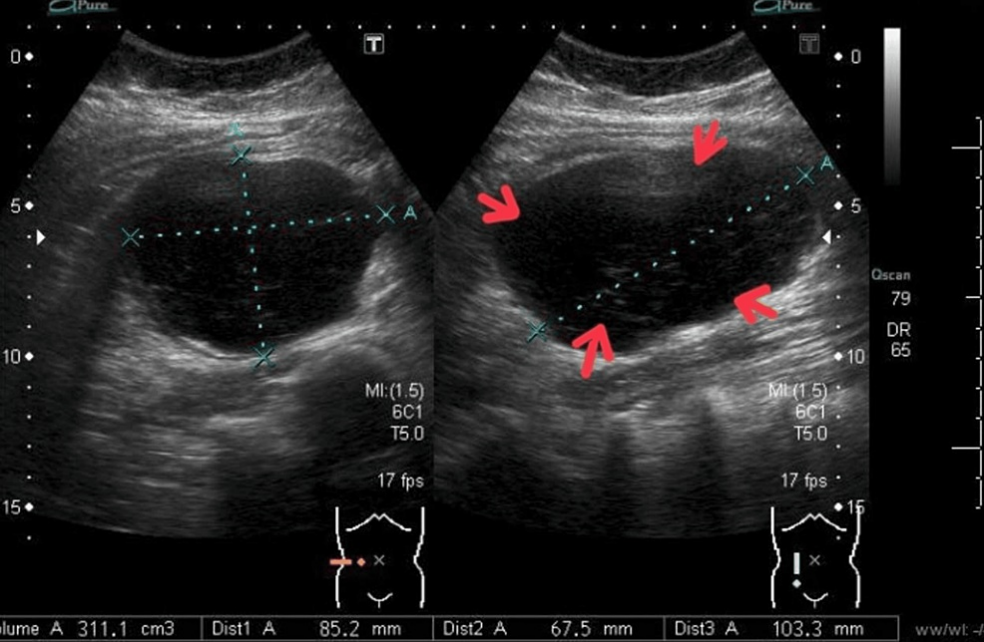
Ascending colon ultrasound revealing a colonic intramural hematoma

The patient was admitted to our hospital for conservative management (stop oral intake, IV fluid D5 1/2 NS 120cc/h for three days, Voltaren 75 mg intramuscular for one day, paracetamol 1 gm intravenous every six hours for three days, Ibuprofen 400 mg every eight hours orally for four days, and omeprazole 40 mg intravenous for eight days. The patient was observed and received antibiotics (metronidazole 500 mg intravenous every eight hours for eight days and cefuroxime 1500 mg every eight hours intravenous for eight days. The patient was started on a fluid and soft diet the following day. She was tolerating an oral diet on the 20th of June 2022, and then a regular diet was initiated three days after the admission. Serial physical examinations were performed throughout the admission which presented a reduction in the size of the abdominal swelling. Abdominal ultrasound was performed as a radiological follow-up on the 23rd of June 2022 which showed mild regression of the size of hematoma 6.7 x 8 x 10 cm (Figure 2).

The patient improved during the period of stay and was stable throughout the admission. The patient was discharged on the 27th of June 2022 in a stable condition and was tolerating an oral diet, passing regular stool, and mobilizing. One month later, the patient presented in the outpatient clinic for follow-up, and she was in good condition with no active complaints.

## Discussion

Gastrointestinal hematomas approximately occur in 0.0004% [5]; the duodenum is the most common site affected, accounting for 93.5% of all gastrointestinal hematomas, while the colon accounts for 4.4% of all gastrointestinal intramural hematomas [5,6]. Diagnosis of bowel hematomas can be clinically challenging due to the nature of the presentation being highly unspecific. It can range from mild abdominal pain to acute abdominal pain, while some present with symptoms of intestinal obstruction with nausea and vomiting. Physical examination reveals rebound pain and abdominal guarding, bloody bowel movements, or signs of intestinal obstruction. Physicians treating intramural hematomas require high levels of suspicion to obtain an early diagnosis and avoid complications [7].

In cases of trauma, ultrasound and X-rays are requested as initial investigations within the primary survey. CT with contrast is the gold-standard investigation to diagnose colonic intramural hematomas. Imaging characteristics include bowel wall thickening, hyper-dense mass, submucosal edema, and luminal expansion; in our case, all of these findings were visualized [8,9].

Colonic intramural hematomas can be approached conservatively if the patient is stable clinically and hemodynamically without any sign of peritonitis, while surgical management is indicated in unstable cases or with the presence of complications such as an acute drop of hemoglobin or an expanding hematoma despite conservative management [8-10].

## Conclusions

Intramural hematoma of the colon needs a high index of suspicion, especially with the presence of variable, unspecific gastrointestinal symptoms. The management of intramural hematoma is case-based. This case demonstrates a colonic intramural hematoma that responded well to conservative management without any complications.
